# Risks and benefits of anti-TNF therapy for ulcerative colitis in a patient with autoimmune hepatitis-related cirrhosis: Case report

**DOI:** 10.1097/MD.0000000000039095

**Published:** 2024-08-02

**Authors:** Renata de Medeiros Dutra, Fernanda Patrícia Jeronymo Pinto, Marcela Maria Silvino Craveiro, Julio Pinheiro Baima, Rogerio Saad-Hossne, Fernando Gomes Romeiro, Ligia Yukie Sassaki

**Affiliations:** a Department of Internal Medicine, São Paulo State University (Unesp), Medical School, Botucatu, Brazil; b Department of Surgery, São Paulo State University (Unesp), Medical School, Botucatu, Brazil.

**Keywords:** anti-TNF therapy, autoimmune hepatitis, case report, liver cirrhosis, ulcerative colitis

## Abstract

**Rationale:**

Ulcerative colitis (UC) is an inflammatory bowel disease (IBD) characterized by continuous inflammation of the colonic mucosa. Autoimmune hepatitis (AIH) is a chronic liver disease characterized by hypergammaglobulinemia, circulating autoantibodies, interface hepatitis, and favorable response to immunosuppression. An association between IBD and AIH is uncommon, and experts have suggested that in patients with overlapping IBD and AIH, the anti-tumor necrosis factor agents can be used. Therefore, this study reports a rare case of a patient with liver cirrhosis due to AIH and UC refractory to conventional treatment and discusses the risks and benefits of using anti-tumor necrosis factor in both conditions.

**Patient concerns:**

A 28-year-old female presented with symptoms of diarrhea, abdominal pain, asthenia, and inappetence, accompanied by abdominal collateral circulation, anemia, alteration of liver enzymes, and elevation of C-reactive protein levels.

**Diagnoses:**

The patient underwent a liver biopsy, which was consistent with liver cirrhosis due to AIH. Colonoscopy showed an inflammatory process throughout the colon, compatible with moderately active UC.

**Interventions:**

The patient received mesalazine, azathioprine, and corticotherapy, with no control of the inflammatory process. Faced with refractoriness to drug treatment and side effects of corticosteroids with an increased risk of severe infection due to cirrhosis, we opted to use infliximab for the treatment of UC. The patient presented with a clinical response and infliximab therapy was maintained.

**Outcomes:**

Eight months after starting infliximab therapy, the patient developed pneumonia with complications from disseminated intravascular coagulation and died.

**Lessons subsections:**

AIH is a rare cause of elevated transaminase levels in patients with UC. The best treatment to control the 2 conditions should be evaluated with vigilance for the side effects of medications, mainly infections, especially in patients with cirrhosis.

## 1. Introduction

Ulcerative colitis (UC) is an inflammatory bowel disease (IBD) that causes continuous inflammation of the colonic mucosa.^[1]^ More than 30% of patients with IBD may have abnormal liver biochemical test results, and primary sclerosing cholangitis (PSC) should be suspected.^[2,3]^ Less commonly, liver diseases not associated with IBD, such as nonalcoholic fatty liver disease, drug-induced liver damage, portal vein thrombosis, liver abscess, autoimmune hepatitis (AIH), overlap syndrome, immunoglobulin (Ig) G4-associated cholangiopathy, hepatic amyloidosis, and granulomatous hepatitis, should be investigated.^[2,3]^

AIH is a chronic liver disease that primarily affects women and is characterized by hypergammaglobulinemia, circulating autoantibodies, association with human leukocyte antigen DR3 or DR4, interface hepatitis on histology, and favorable response to immunosuppression.^[4]^ The diagnosis is confirmed by the clinical picture (fatigue, asthenia, arthralgia, or symptoms related to other associated immune diseases such as IBD), the elevation of aspartate aminotransferase/alanine aminotransferase, IgG, autoantibodies such as antinuclear antibodies (ANAs) and anti-liver kidney microsome type 1, and histological changes.^[5]^

An association between IBD and AIH is uncommon.^[6]^ In a review carried out by Perdigoto et al (1992),^[7]^ the association between UC and AIH was noted in 16% of the 105 evaluated patients. Among patients with IBD in a reference center in Brazil, 0.7% of AIH cases were found in patients with Crohn disease.^[8]^

Experts have suggested that patients with overlapping IBD and AIH are more prone to relapse and progression to cirrhosis, and anti-tumor necrosis factor (TNF) agents can be used in patients with IBD who also have liver disease.^[7,9]^ However, the challenge in treating both conditions with immunosuppressive drugs or biological therapy is the risk of developing serious infections associated with advanced liver disease.^[10]^ Considering the rarity of the case and its implications for therapeutic management, this study aims to present a case of a patient with liver cirrhosis due to AIH and UC refractory to conventional treatment and discuss the risks and benefits of using anti-TNF therapy for these conditions.

## 2. Case presentation

A 28-year-old female was admitted to the hospital in July 2016, with a history of diarrhea, 5 to 6 bowel movements per day, liquid stools without blood or mucus, associated with diffuse abdominal pain, asthenia, and lack of appetite for 9 months. Physical examination revealed collateral circulation in the abdomen. Laboratory tests showed changes in liver enzymes, anemia, and pro-inflammatory markers (Table 1). Serological tests for chronic viral hepatitis were negative, and ANA levels were 1/320 units/EU. A liver biopsy revealed active chronic hepatitis, stage 4 lymphohistiocytic infiltrate, and piecemeal necrosis. The patient was diagnosed with AIH based on the International Autoimmune Hepatitis Group score (11 points), and liver cirrhosis. Ultrasonography showed signs of chronic liver disease and portal hypertension, upper digestive endoscopy showed medium- to large-caliber esophageal varices, and rubber band ligation was performed.

**Table 1:** Evolution of the patient’s biochemical test results and drug treatment.

	Reference value	July 06, 2016	November 29, 2016	May 16, 2017	July 25, 2017	June 15, 2018	March 22, 2019	August 26, 2019	October 17, 2020	February 23, 2021	July 22, 2021	October 24, 2021
Hemoglobin (g/dL)	12–16	6.9	6.6	5.5	6.7	7.2	7.4	5.3	8.7	6.1	4.1	5.9
Hematocrit (%)	3646	23	22.1	18.3	22.3	24.3	25.8	19.2	28.5	21.0	15.1	19.6
Platelets (mm^3^)	140,000–440,000	172.000	131.000	142.000	92.000	121.000	102.000	124.000	226.000	116.000	93.000	60.000
Leukocytes (mm^3^)	4000–11,000	2.800	2.000	2.700	2.000	1.900	3.800	2.700	13.200	1.800	3.700	5.900
C-reactive protein (mg/dL)	<1	2.8	-	1.6	0.9	1.7	-	4.1	6.2	1.2	5.1	18.8
AST/ALT (U/L)	14–36/12–43	109/49	84/45	210/93	54/50	183/89	31/20	37/21	41/36	61/38	35/27	41/23
Total bilirubin	0.2–1.3	1.8	1.15	1.6	1.2	1.4	1.4	2.0	2.0	2.5	2.2	5.2
ALP/GGT (U/L)	36–126/12–43	327/585	165/273	305/497	126/362	222/340	163/47	246/264	249/358	174/178	264/319	116/163
Medications	-	Hospital admission. Ciprofloxacin, azathioprine, and prednisone (irregular use)	Azathioprine and prednisone	Azathioprine suspension, use of prednisone 45 mg/d + mesalazine 4 g/d	Azathioprine 100 mg/d and mesalazine 4 g/d	Azathioprine suspension, prednisone 60 mg/d, and mesalazine 4 g/d	Pregnancy, mesalazine 4 g/d	Prednisone 60 mg/d, mesalazine 4 g/d	Prednisone 60 mg/d, mesalazine 4 g/d	Infliximab 2nd dose of induction	Infliximab maintenance	Infliximab maintenance death

ALP = alkaline phosphatase, ALT = alanine aminotransferase, AST = aspartate aminotransferase, GGT = gamma-glutamyl transferase.

Azathioprine and prednisone were initiated in July 2016 for AIH treatment. In March 2017, she presented with ascites and worsening pancytopenia, and azathioprine was discontinued. However, she also presented with bloody diarrhea and abdominal pain. Faced with the return of intestinal symptoms, she underwent a colonoscopy for investigation, and the colonoscopic lesions were consistent with UC with moderate activity. Treatment with low-dose azathioprine was restarted with mesalazine (4 g/day) and the patient showed a clinical response. The patient underwent a second liver biopsy that maintained the characteristics of the first biopsy, with no histological pattern of overlap with PSC.

**Figure 1.**
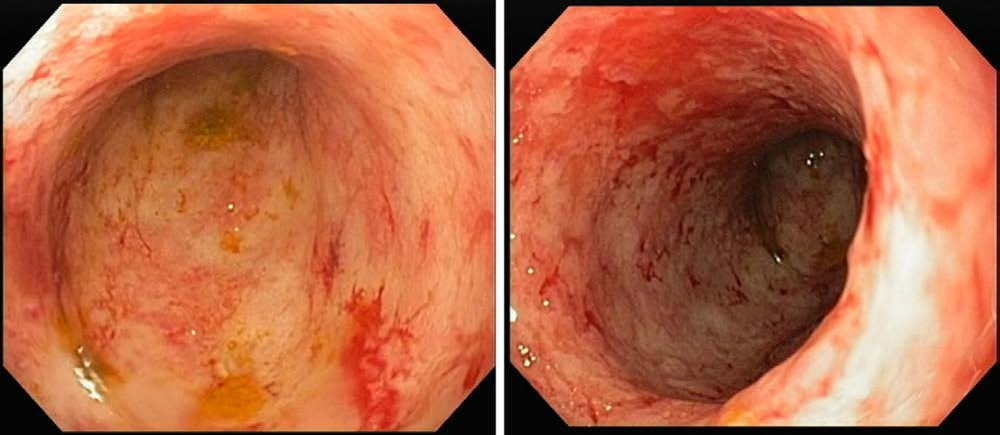
Sigmoidoscopy images showing severe ulcerative colitis, mucosal edema and erythema, friability, and spontaneous bleeding, compatible with a Mayo endoscopic score of 3.

Between 2017 and 2018, the patient had periods of clinical UC activity requiring corticosteroid use, and azathioprine was discontinued because of worsening pancytopenia. In July 2018, the patient was asymptomatic and pregnant. She was maintained on mesalazine. In March 2019, she underwent cesarean delivery and was still maintained on mesalazine. In July and August 2019, she required hospital admission due to UC activity, as evidenced by sigmoidoscopy, with lesions compatible with severe active pancolitis (Mayo endoscopic score 3) (Fig. 1). The patient was treated with oral prednisone (60 mg/day), with improvement in intestinal symptoms.

In 2020, she presented with UC recurrence, bloody diarrhea, fever, asthenia, and decompensation of liver cirrhosis. She required blood transfusion, antibiotic therapy, and the use of vasoactive drugs. The patient continued using mesalazine 4 g/day and diuretics (furosemide and spironolactone) to treat ascites. In September 2020, she was hospitalized with abdominal septic shock. She was discharged on mesalazine and prednisone (60 mg/day), and diuretics were continued. Sigmoidoscopy showed mild UC activity (Mayo endoscopic score 1) and upper digestive endoscopy revealed esophageal moniliasis (Kodsi III) which was treated with fluconazole for 14 days.

Due to multiple hospitalizations with refractoriness to treatment with mesalazine, hematological side effects of azathioprine, side effects of corticosteroid use, and the risk of serious infection in patients with cirrhosis, we opted for the use of infliximab, an anti-TNF biological therapy. This was considered because the patient had no liver transplantation scheduled due to low therapeutic adherence and no family support to ensure posttransplantation follow-up. After evaluating the risks and benefits with the hepatology team, induction therapy with the drug was started in February 2021, with symptomatic improvement. Colonoscopy showed UC with mild activity (Mayo endoscopic score of 1). Biochemical tests showed normalization of the transaminase levels (Table 1).

**Figure 2.**
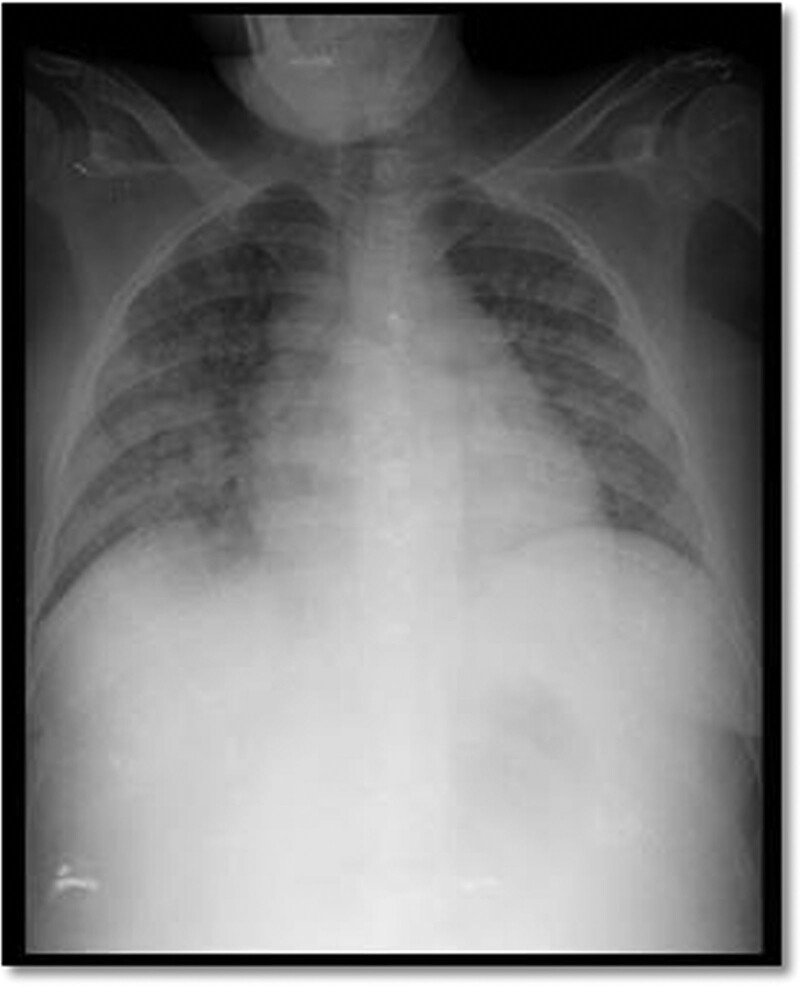
Chest radiograph showing pulmonary bilateral diffuse infiltrates.

In October 2021, the patient was hospitalized with pneumonia evidenced by diffuse pulmonary infiltrates on chest radiography (Fig. 2) and received antibiotic therapy with ceftriaxone and azithromycin, in addition to blood transfusions. The patient developed worsening liver function and disseminated intravascular coagulation. Despite treatment, the patient developed acute respiratory failure and cardiorespiratory arrest 24 hours after hospital admission.

The study was approved by the local (São Paulo State University - Unesp) Research Ethics Committee and the consent form was waived, since the patient died, with the authors being responsible for the patient’s anonymity (CAAE: 52623721.0.0000.5411). The written informed consent was not secured from the legal guardian/next of kin for patients within this specific age group.

## 3. Discussion

In this case report, we described a patient with liver cirrhosis due to AIH and UC refractory to conventional therapy who had fatal complications related to the infection due to immunosuppression. Despite the risks of immunosuppression, patients need to be treated for their underlying disease to avoid the risk of complications, always weighing the risk and benefits of immunosuppressive therapy in critical patients.

AIH is a relatively rare liver disease of unknown etiology and global distribution that affects all age groups, ethnic groups, and both sexes. The diagnosis is based on clinicopathological features, such as polyclonal hypergammaglobulinemia, particularly IgG, circulating autoantibodies, interface hepatitis, absence of viral hepatitis, and favorable response to immunosuppression.^[4]^

The guidelines of the American Association for the Study of Liver Disease recommend treatment with prednisone 60 mg/day or a combination of prednisone 30 mg/day and azathioprine 50 mg/day, with the latter favored as the initial treatment due to the lower frequency of side effects. As a second-line treatment, the use of agents such as cyclophosphamide, methotrexate, rituximab, or infliximab is recommended.^[5,11]^ Liver transplantation is the definitive treatment for patients with AIH and cirrhosis.^[12]^ PSC and AIH are the main causes of liver transplantation in patients with IBD, and relapse of the disease occurs in 25% of cases.^[12,13]^ Poor adherence to treatment and posttransplant follow-up were contraindications for performing the procedure in this case.

Although infliximab is mentioned as an alternative therapy for AIH, there has been heterogeneity in the studied population. Furthermore, studies aimed at treating IBD as the main disease did not allow the evaluation of the real benefit of using anti-TNF agents for AIH.^[11]^ The weak level of evidence and increased risk of infection, especially in patients with cirrhosis, do not justify the use of anti-TNF agents as a second-line treatment in this specific population. However, the decision is based on local experience, the severity of AIH, and patient characteristics. Weiler-Normann et al (2013)^[14]^ reported the first case series of 11 patients with refractory AIH, 7 of whom were diagnosed with liver cirrhosis. Treatment failure also occurred in these patients, and they received infliximab 5 mg/kg. After induction therapy, all patients showed a decrease in IgG levels and liver enzymes, with normalization of tests in 8 patients and improvement in histological activity in 5 patients. Although the results were promising, the medication should be used with caution owing to the risk of drug-induced AIH in patients with extrahepatic autoimmune diseases, as well as the risk of serious infections. In this report, 7 patients had infectious complications such as ocular herpes, recurrent urinary tract infection, and pneumonia. Therefore, the use of infliximab is limited to patients with advanced liver disease, such as the patient presented in this case report.

Azathioprine is associated with increased adverse events, such as leukopenia, thrombocytopenia, pancreatitis, hepatitis, opportunistic infections, arthralgia, lymphoma, and nonmelanoma skin cancer.^[15,16]^ It is also known that corticosteroids, used as part of the first-line treatment of AIH, are associated with a dose- and duration-dependent risk of infection. These infections include tuberculosis, hepatitis B, and *Strongyloides stercoralis* and *Pneumocystis jirovecii* pneumonia. Screening and antimicrobial prophylaxis may be indicated in patients treated chronically (≥8 weeks of continuous or intermittent corticosteroid use) with moderate doses (≥15 mg to <30 mg).^[17]^

Patients with cirrhosis are at risk of complications, such as the development of infections, high morbidity and mortality, and frequent hospital admissions.^[10]^ The term cirrhosis-associated immune dysfunction refers to the dynamic spectrum of immune disturbances that develop in patients with cirrhosis, which are closely linked to underlying liver disease and negatively correlated with prognosis. At both ends of the cirrhosis-associated immune dysfunction spectrum, systemic inflammation can exacerbate the clinical manifestations of cirrhosis, such as hemodynamic disturbances, kidney damage, and immunodeficiency, which contribute to the high rate of infection in patients with decompensated cirrhosis. Therefore, the use of immunosuppressants in patients with cirrhosis should be evaluated on a case-by-case basis, always balancing the risks and benefits. However, despite the risk of immunosuppression in patients with cirrhosis with IBD, disease remission should be sought to avoid decompensation of cirrhosis.

Severe hepatotoxicity is a very rare condition during biological therapy and is more frequent in patients treated with infliximab.^[3,18]^ In a study involving 3340 patients using infliximab as a therapy for Crohn disease over 13 years, hepatotoxicity and hepatitis emerged as adverse events in 5 patients (0.01/100 patient-years).^[19]^

Therefore, IBD can overlap with other autoimmune diseases such as AIH. Despite the low level of evidence, and the limitation of this study witch describes a single case, and requires controlled studies with an adequate number of patients in order to define the risks and benefits of using anti-TNF in overlapping cases, it is important to present cases with good prognosis using anti-TNF as a therapeutic option for the treatment of both conditions, and monitoring side effects that may be more serious in patients with cirrhosis, as reported in this case.

AIH is a rare cause of elevated transaminase levels in patients with UC. This condition can progress to liver cirrhosis and is associated with complications related to immune dysfunction. Thus, the best treatment that can control both conditions should be evaluated, especially in patients with cirrhosis, who should continuously monitor their possible side effects. The main complications are infections, and adequate surveillance of this at-risk population must be rigorous, to control underlying conditions and ensure patient safety.

## Author contributions

**Conceptualization:** Ligia Yukie Sassaki.

**Formal analysis:** Renata de Medeiros Dutra.

**Supervision:** Julio Pinheiro Baima, Fernando Gomes Romeiro, Ligia Yukie Sassaki.

**Visualization:** Fernanda Patrícia Jeronymo Pinto, Marcela Maria Silvino Craveiro, Rogerio Saad-Hossne.

**Writing – original draft:** Renata de Medeiros Dutra.

**Writing – review & editing:** Renata de Medeiros Dutra, Ligia Yukie Sassaki.
